# The Influence of an Eight-Week Home Exercise Program on Spatiotemporal and Kinetic Characteristics of Gait and Knee Function in Women with Severe Knee Osteoarthritis Scheduled for Arthroplasty

**DOI:** 10.3390/medicina61050774

**Published:** 2025-04-22

**Authors:** Monika Mets, Jelena Sokk, Jaan Ereline, Mati Pääsuke, Tiit Haviko, Helena Gapeyeva

**Affiliations:** 1 Institute of Sport Sciences and Physiotherapy, University of Tartu, Ujula 4, 51008 Tartu, Estonia; jelena.sokk@gmail.com (J.S.); jaan.ereline@ut.ee (J.E.); mati.paasuke@ut.ee (M.P.); helena.gapeyeva@itk.ee (H.G.); 2 Physiotherapy and Environmental Health Department, Tartu Applied Health Sciences University, Nooruse 5, 50411 Tartu, Estonia; 3 Department of Traumatology and Orthopaedics, Institute of Clinical Medicine, University of Tartu, L. Puusepa 8, 51014 Tartu, Estonia; havikotiit@gmail.com; 4 II Rehabilitation Department, Clinic of Medical Rehabilitation, East Tallinn Central Hospital, Pärnu 104, 11312 Tallinn, Estonia

**Keywords:** gait, home exercise program, knee osteoarthritis, preoperative rehabilitation, extension moment, health status

## Abstract

*Background and Objectives:* The increased prevalence of knee osteoarthritis (OA) and need for total knee arthroplasty (TKA) indicate a growing need for effective prehabilitation. The effect of preoperative home exercise programs (HEPs) on gait in patients with severe knee OA is under-investigated. This study aimed to evaluate the influence of an 8-week preoperative HEP on gait characteristics, leg extensor muscle strength, knee function, and health status in women with severe knee OA scheduled for TKA and to compare them with healthy control data. *Material and Methods:* Eighteen women with severe knee OA (KOA, aged 61.8 ± 1.6 years) and ten age-matched healthy women (CON) participated in this study. The KOA group performed an HEP with 15 exercises aimed at improving lower limb muscle strength, motion, balance, and coordination. Gait spatiotemporal and kinetic characteristics during the loading response, isometric leg extensor strength, knee active range of motion (AROM), and The Western Ontario and McMaster Universities Arthritis Index (WOMAC) were investigated. Associations between characteristics were analyzed. *Results:* Improvements in ground reaction force (GRF) during the loading response of gait, leg extensor muscle strength, the knee AROM, and the WOMAC index were found post-HEP. The KOA group demonstrated lower (*p* < 0.05) spatiotemporal and GRF characteristics than the CON group. Knee extension moment (KEM) was lower pre-HEP (*p* < 0.05) but did not differ significantly from the CON group post-HEP. Gait characteristics and WOMAC were associated with leg extensor muscle strength and knee AROM and pain in the KOA group. *Conclusions*: An eight-week preoperative HEP improved GRF and KEM during the loading response of gait, muscle strength, knee function, and self-reported knee OA-related health status in women with severe knee OA. Preoperative HEP before TKA, focusing on leg extensor muscle strength, range of motion, and pain relief, is an effective alternative to supervised exercise therapy in women with severe knee OA.

## 1. Introduction

Osteoarthritis (OA) of the knee accounts for over 80% of the general OA burden [1]. Due to population aging, the prevalence of knee OA is becoming one of the leading causes of disability in the world [2,3]. Along with that, the need for total knee arthroplasty (TKA) is increasing and leading to higher financial burden [2]. Over 364 million people worldwide experience activity limitations caused by OA, and women are more affected by knee OA and tend to experience knee OA symptoms greater than men [4,5,6]. In Germany, 2/3 of TKAs are performed on women [7]. The main knee OA symptoms are joint pain, stiffness, reduced range of motion, muscle weakness, and altered gait patterns, along with decreased quality of life and self-reported health status. OA causes significant difficulties in performing activities of daily living or even cessation of valued daily activities, such as walking for exercise [5,8,9,10,11]. Walking is closely associated with quality of life [12], and gait is an important indicator of health and function in older adults, predicting or revealing motor decline [13]. Hip and knee osteoarthritis are common non-neurological causes of gait disorders, often leading to increased risk of falling and lower quality of life and independence [14,15].

Three-dimensional instrumented gait analysis is used to assess normal and pathological gait in clinical practice and research to determine functional limitations related to pathologies or effects of implemented rehabilitation programs aimed to reduce those limitations [16]. It can be a measure of knee OA severity, symptoms, and everyday function [17]. Three-dimensional instrumented gait analysis is used as a gold standard to measure temporal parameters (e.g., velocity, cadence), kinematics (joint motion), and kinetics (e.g., ground reaction force, joint moments). Spatiotemporal characteristics are essential descriptors of human gait and are often used to assess function and mobility in clinical practice. Joint moments during gait cycle stance and swing phases are particularly informative in evaluating patients with joint problems such as osteoarthritis [15,16,18]. However, joint moments and ground reaction forces are generally the least studied compared to other gait characteristics such as spatiotemporal [12].

Knee OA patients demonstrate changes in spatiotemporal, kinetic, and kinematic gait characteristics, mainly to avoid pain and joint loading [19]. Walking speed is reduced and tends to become slower as the disease progresses [20]. Decreased stance phase knee flexion angles and early stance phase knee extension moments have also been found and tend to worsen with the progression of knee OA [20]. These changes are likely related to symptoms such as pain worsening as knee OA progresses. Fukaya et al. [19] observed a more significant reduction in knee extensor moment in the limb, which was reported as more painful by patients with severe knee OA. Another reason could be weakness of lower limb muscles. The main muscles working during the early stance phase are knee extensors, including quadriceps, and knee flexors, including hamstrings [21]. These muscle groups are also commonly most affected by knee OA, and the decreased knee extensors/flexors strength are also a risk factor for knee OA progression [11,22,23].

Gait could also be affected by joint stiffness, which is a coping mechanism for dealing with joint instability [24]. The Western Ontario and McMaster University Osteoarthritis (WOMAC) index is a widespread patient-reported outcome measure to determine the severity of knee OA based on pain, stiffness, and function [25]. Gait characteristics are significantly associated with self-reported outcome measures [17]. Therefore, patient self-reported measures, including WOMAC and VAS, should be included in planning treatments and evaluating their effects [26].

It is known that regular physical activity helps the symptoms of knee OA. Therefore, it is crucial to implement exercise therapy to enhance patient outcomes. Patient education and exercise therapy are beneficial and safe for knee OA patients both before and after TKA [27]. Based on recommendations, exercise therapy should include aerobic exercises, strengthening exercises (e.g., resistance training with elastic bands), neuromuscular training, and balance training [27,28,29,30], which have been found to reduce pain and improve physical function and self-reported health status in knee OA patients [28,31,32]. However, post-TKA interventions tend to be more widely researched. There is a need for studies investigating different preoperative exercise treatments [33], mainly because better preoperative health status contributes to enhanced postoperative recovery after TKA [34,35,36]. Preoperative exercise therapy shows promising results, and even as little as 3 weeks of preoperative rehabilitation aimed to increase lower limb muscle strength prior to TKA has shown significant improvement in physical activity, knee ROM, and pain [36]. However, inpatient rehabilitation may not always be suitable or available. Here, a preoperative home exercise program (HEP) offers an alternative when clinical or financial options are limited [37,38].

Although supervised exercise programs are more recommended than HEPs [30,33], the latter offer good-quality treatment. HEPs show similar results to outpatient exercise or medical treatment, and the effect of HEPs on the complications of knee OA needs further investigation [38]. HEPs can improve knee pain, muscle strength, knee AROM, gait speed and patterns, and self-reported health status and quality of life in knee OA patients. The average duration of these HEPs is 6–12 weeks [33,37,38,39,40,41]. Also, since regular exercising is essential for improving function, pain, and quality of life, exercise therapy should be tailored to be as accessible as possible [31]. HEPs can make exercise therapy more accessible and are a safe, easy-to-use, time-effective, and inexpensive supplement to outpatient programs for knee OA patients [38,42,43]. Taking part in a preferred exercise therapy option may increase persistent exercise performance, as patients are unlikely to participate if participation is difficult due to lack of transportation or funds [30,44].

Despite that HEPs are effective and suitable for knee OA patients [45,46,47], the number of studies is small compared to supervised exercise therapy, especially regarding patients with severe knee OA scheduled for TKA. Previous investigations on preoperative exercise therapy have primarily focused on patients with mild-to-moderate knee OA [33,37,40,46], with fewer concentrating on prehabilitation in patients with severe knee OA [34,36,48,49], though HEP is effective and could even possibly postpone TKA [50].

Based on previous studies, more research is needed on the effect of preoperative HEPs on gait characteristics and associations between gait characteristics and other widespread outcome measures in patients with severe knee OA. This study aimed to evaluate the effect of an 8-week preoperative HEP on spatiotemporal and kinetic gait characteristics, alongside leg extensor muscle strength, knee function, and OA-related health status, in women with severe knee OA scheduled for TKA, and to compare data with healthy age- and gender-matched controls. Regarding the aim, three hypotheses/research questions were proposed: (1) performing an 8-week preoperative HEP improves gait spatiotemporal and kinetic characteristics during the loading response, as well as self-reported knee OA-related health status in women with severe knee OA prior to TKA; (2) do measured characteristics in the KOA group improve to the CON group level post-HEP?; (3) spatiotemporal and kinetic characteristics of gait and self-reported knee OA-related health status are associated with lower limb muscle strength, knee joint range of motion, and knee pain in women with severe knee OA undergoing TKA.

## 2. Materials and Methods

### 2.1. Study Design

This longitudinal pre–post-experimental comparative study was conducted following the Declaration of Helsinki and was approved by the Research Ethics Committee of the University of Tartu, Tartu, Estonia (protocols number 153/9 and 218/T-16, dates of approval 16 October 2006 and 24 September 2012), and was registered with ClinicalTrials.gov (registration number NCT02881463). The study was conducted by researchers in the Laboratory of Kinesiology and Biomechanics of the University of Tartu in cooperation with the Department of Traumatology and Orthopaedics, Institute of Clinical Medicine of the University of Tartu, during the period of 2011–2014. The rights of the participants were protected. All participants signed written consent to participate in the study voluntarily. All mandatory laboratory occupational health and safety requirements were followed during the study.

### 2.2. Participants

Twenty-eight women aged 46–72 years participated in this study: patients with knee OA (n = 18, KOA group) and age-matched healthy controls (n = 10, CON group). The data of participants are shown in Table 1.

The same orthopedic surgeon recruited the KOA group and assessed the severity of the knee OA via X-ray [51]. All KOA group participants were scheduled for primary TKA and evaluated twice—before and after performing the 8-week preoperative HEP. The criteria for inclusion in the KOA group were knee OA stage III or IV in regard to X-ray change according to the Kellgren and Lawrence classification [51], assignment of a primary unilateral TKA, unassisted gait, and motivation to exercise at home daily for eight weeks.

The CON group was included in the study to establish a baseline of functional data. This baseline allowed the evaluation of the HEP’s effect in the KOA group by comparing the KOA group’s data against the CON group’s data before and after the implementation of HEP in the KOA group (pre-HEP and post-HEP, respectively). The CON group was age-matched to the KOA group and consisted of ten healthy women with no evidence of knee OA, as confirmed via X-ray evaluations conducted by the same orthopedic surgeon responsible for recruiting the KOA group. Participants in the CON group were recruited from the community by the same physiotherapist and participated in the study once. The inclusion criteria for the CON group were no evidence of radiological knee OA and no participation in any regular training (the latter was verified via interview).

To reduce influencing factors, the exclusion criteria for both groups included a history of major trauma or surgery of the lower limbs (including joint arthroplasty), as well as other neurological, musculoskeletal, pulmonary, and cardiovascular conditions or pathologies that could influence the measurement outcomes, including disturbances in body balance and coordination. The assessments were conducted in the mornings, and participants were instructed not to take pain-relieving medication before the assessments.

### 2.3. Preoperative Home Exercise Program

The KOA group performed a preoperative HEP daily for eight weeks. The HEP consisted of 15 exercises designed to enhance muscle strength, range of motion, body balance, and coordination, especially targeting lower limbs [52], including standing and seated exercises that are easy for patients with severe knee OA to perform independently in a home environment. The progression, sets, repetitions, and descriptions of the exercises can be found in Appendix A. A physiotherapist selected the exercises, which were the same for all participants in the KOA group. Before the training period, the physiotherapist introduced and taught the exercises to each KOA group participant individually to ensure proper performance, and weekly follow-ups concerning knee joint status and the HEP were made over the phone by the same physiotherapist, as it has been previously found that instruction on exercises and follow-up calls increase the effect of HEPs [40,53]. These phone calls also offered participants a chance to seek feedback, ask questions, and receive guidance on exercise techniques.

The participants in the KOA group were provided with a set of elastic bands featuring three resistance levels. Resistance was monitored throughout the HEP using these elastic bands with predefined resistance levels (green, red, and blue) based on Theraband (USA) manufacturer specifications. Participants were instructed to progress to a higher resistance band as exercises became easier, with additional structured guidelines for progression at specific weeks (Appendix A). The elastic bands were used in various seated exercises to provide resistance, targeting the lower limbs. The KOA group also received an illustrated handout of the HEP with descriptions and progression recommendations for each week. Research has shown that printed exercise manuals in knee OA patients can increase the effectiveness and benefits of HEPs performed without constant supervision, and higher weekly frequency of HEP performance correlates with improved outcomes [46,54].

The KOA group also filled out a training diary to mark down information regarding the HEP (e.g., time exercised, other activities, pain, etc.). All KOA group participants completed the HEP (dropout n = 0). After performing the 8-week HEP, 13 of the 18 participants in the KOA group underwent TKA, while 5 chose to postpone the surgery.

### 2.4. Gait Characteristics Measurement

Gait characteristics were recorded by an instrumented 3D gait analysis system, a method proven suitable for describing gait changes in patients with knee OA [55]. In the present study, a 3D optic–electronic movement analysis system (ELITE, BTS SpA, Garbagnate Milanese, MI, Italy), which included six infrared cameras (sampling rate 100 Hz), was used to measure sagittal kinetic and spatiotemporal gait characteristics. Two force platforms (sampling rate 500 Hz) (Kistler 9286A, Winterthur, Switzerland) were used to assess ground reaction force (GRF).

Reflective markers were attached on selected anatomical points, according to the Helen Hayes protocol using the Davis biomechanical model: processus spinosus of C7 and L4, acromion, anterior superior iliac spines, lateral femoral trochanters, lateral mid-thigh, lateral femoral epicondyle, lateral tibial epicondyle, lateral mid-shank, lateral malleolus, lateral side of the foot on the 5th metatarsal head, and calcaneus [56]. The participant walked along a 5.8 m walkway at a self-selected comfortable speed during the measurement. A maximum of five trials were measured, and the trial where the walking speed had the lowest SD was considered for further analysis.

Gait measurement data were stored and analyzed using ELITE Clinic software, version 3.4.106 (BTS SpA, Garbagnate Milanese, MI, Italy). The following data were considered for further analysis: spatiotemporal parameters—stance time (%), cadence (steps/min), mean velocity (m/s), step length (m), stride length (m), and step width (m); and sagittal kinetic parameters—knee extension moment (N·m/kg) and vertical GRF (N) during the loading response of gait. GRF and KEM data were normalized to each participant’s body mass.

### 2.5. Leg Extensor Muscle Strength Measurement

Investigation of lower limb strength using closed-chain assessment, rather than open-chain knee extensor strength, may provide a clearer understanding of functional limitations in knee OA patients [57]. For the assessment of isometric maximal voluntary contraction (MVC) force (hereafter, leg extensor muscle strength), the participant was seated on a horizontal dynamometric chair with their hip joints equal to 120 degrees [58]. The participant’s position was secured with a Velcro belt over the pelvis. The participant’s feet were placed on a footplate connected to a standard strain gauge transducer (1778 DST-2, Russia). The participant was asked to push the footplate with maximal effort when the signal light switched on and to relax when it switched off. The signal light was on for two seconds. The allowed resting period between trials was two minutes. The rate of force development (RFD, N/s) was recorded at 50% of MVC, and the relative peak torque development (RPTD) (N·m·s^−1^·kg^−1^) was calculated. Three trials were performed, and the highest result was taken into consideration.

### 2.6. Active Knee Range of Motion Measurement

The active knee range of motion (flexion and extension) was measured with a long-arm goniometer (Baseline, USA). Goniometers are reliable and accurate for assessing knee joint range of motion [59]. The active knee flexion range of motion (AROM flex) was measured in the prone position, and the active extension range of motion (AROM ext) was measured in the supine position. The goniometer center was placed in the middle of the femoral lateral epicondyle, the mobile arm parallel to the fibula and the stationary arm parallel to the femur. The participant was asked to flex or extend their knee joint maximally. Pelvic tilting was prohibited. Three measurements were taken, and the best result was considered for analysis.

### 2.7. Timed Up and Go Test Measurement

Dynamic balance, lower extremity function, and functional mobility were measured with the Timed Up and Go (TUG) test, which is a widely used sensitive, reliable, and valid method in patients with orthopedic complications and various interventions [60,61]. The participant sat on a chair with a marker placed three meters from the chair. On command, the participant stood up, walked around the marker, and returned to the seat. The timer was stopped when the participant was fully seated. One practice trial was performed before the measurement. The test was performed twice, with a rest time of two minutes, and the best result was taken for analysis.

### 2.8. Self-Reported Knee OA-Related Health Status Scoring

Self-reported health status was studied with the Western Ontario and McMaster Universities Osteoarthritis Index (WOMAC), a valid, reliable, and efficient lower limb OA-specific outcome measure [62,63]. The questionnaire consisted of 24 questions divided into three subscales: pain (5 questions), stiffness (2 questions), and function (17 questions), which the participants answered on a 5-point Likert response scale. For better interpretation and comparison, the WOMAC score was converted by a scoring algorithm (WOMAC index = score × 100/96) and analyzed on a scale of 0–100, with higher scores reflecting better health status [63].

Knee pain was assessed with the visual analogue scale (VAS), which is a reliable pain measure in OA patients [64]. Knee pain was estimated on a 100 mm line, where 10 mm was equal to 1 point, with 0 points = no pain and 10 points = maximum pain. The participants were instructed to evaluate knee pain experienced during the last seven days [65].

### 2.9. Statistics

Data in the KOA group were analyzed as the involved limb (scheduled for TKA) and uninvolved limb (contralateral, not scheduled for TKA), and in the CON group, as data of the dominant limb for comparison. Data are means and standard errors (SEs). A power analysis showed that a sample size of 18 is sufficient to give the study a 95% confidence level to detect an effect of HEP on gait characteristics in the KOA group. For the assessment of the normality of data distribution, the Shapiro–Wilks test was used.

Statistics were calculated using the SPSS 20 (IBM, Armonk, NY, USA). A one-way analysis of variance (ANOVA) followed by the independent sample *t*-test was used to evaluate differences between the two groups, as well as between the involved and uninvolved limb in the KOA group, and the paired sample *t*-test was used for differences in the KOA group’s pre- and post-HEP results. The Pearson correlation coefficients were found to assess associations between variables to further analyze the HEP’s effect. The clinical effect size was calculated as Cohen’s *d* to assess inter-group clinical relevance, and differences were considered clinically significant when CES ≥ 0.4 [66]. Statistical significance was set at *p* < 0.05.

## 3. Results

### 3.1. Gait Data 

Spatiotemporal characteristics did not change statistically significantly (*p* > 0.05, CES was 0.01–0.33) in the KOA group post-HEP compared to pre-HEP. The KOA group demonstrated significantly poorer results (*p* < 0,05) in all spatiotemporal characteristics, except for step width both pre-HEP and post-HEP, when compared to the CON group (Table 2).

**Figure 1:**
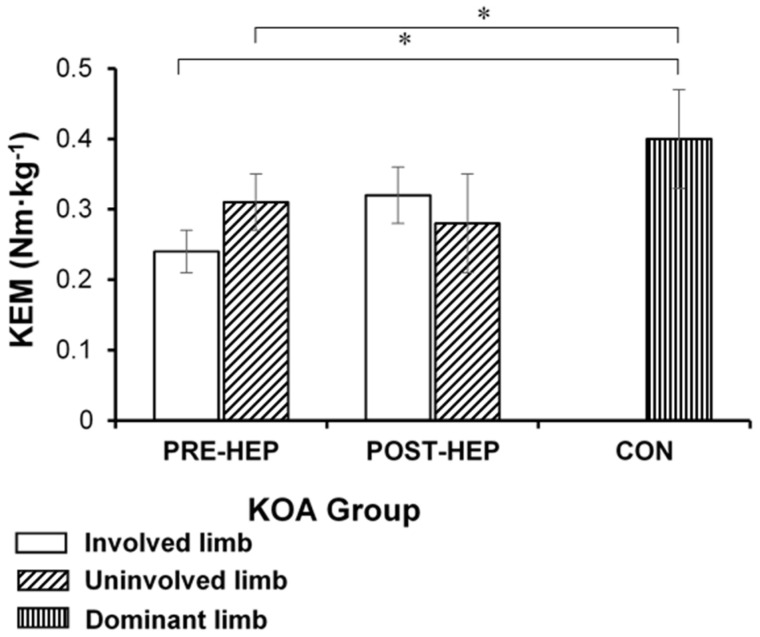
Knee extension moment (KEM) during the loading response of gait (mean and SE) in women with severe knee osteoarthritis (KOA) before and after a home exercise program (HEP) compared to healthy controls (CON). Involved limb—leg scheduled for TKA in the KOA group; uninvolved limb—other leg in the KOA group; dominant limb—dominant leg in the CON group; * *p* < 0.05.

KEM of the involved limb, analyzed during the loading response of gait, did not change significantly (*p* > 0.05, CES 0.23) post-HEP compared to pre-HEP in the KOA group. However, comparing the KOA group’s involved limb’s KEM with the CON group’s dominant limb, the KOA group’s result was significantly lower pre-HEP (*p* < 0.05) but not post-HEP (*p* > 0.05) (Figure 1).

**Figure 2:**
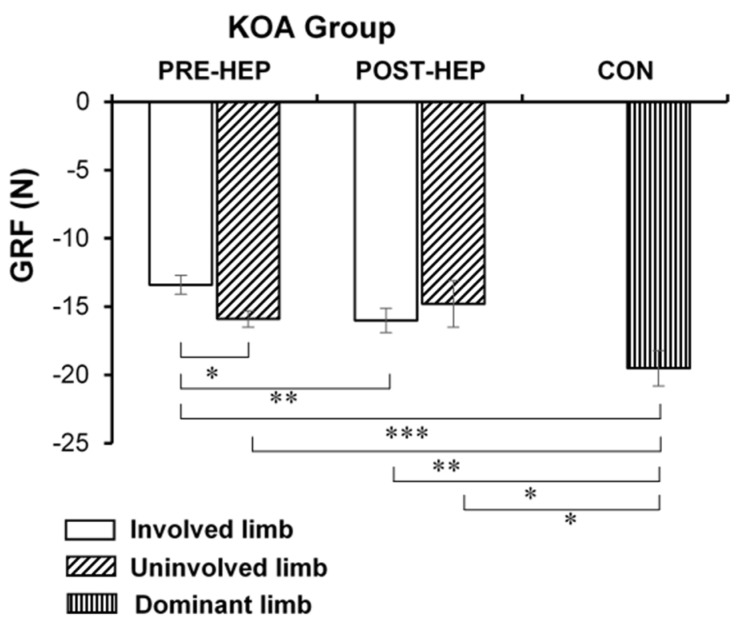
Ground reaction force (GRF) during the loading response of gait (mean and SE) in women with severe knee osteoarthritis (KOA) before and after a home exercise program (HEP) compared to healthy controls (CON). Involved limb—leg scheduled for TKA in the KOA group; uninvolved limb—other leg in the KOA group; dominant—dominant leg in the CON group; * *p* < 0.05; ** *p* < 0.01, *** *p* < 0.001.

GRF, analyzed during the loading response of gait (Figure 2), increased (*p* < 0.01, CES = 0.35) in the KOA group’s involved limb post-HEP compared to pre-HEP. GRF was lower in the involved limb (*p* < 0.05) compared to the uninvolved limb pre-HEP but not post-HEP (*p* > 0.05). The GRF of the involved limb in the KOA group was lower pre-HEP (*p* < 0.001) and post-HEP (*p* < 0.05) compared to the CON group’s dominant limb.

### 3.2. Leg Extensor Muscle Strength

**Figure 3:**
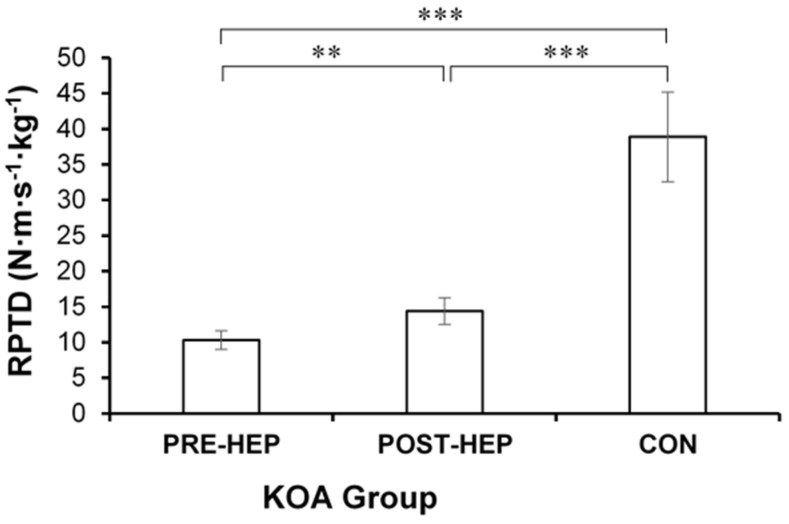
Relative peak torque development (RPTD) of leg extensor muscles at 50% maximum voluntary contraction force (mean and SE) in women with severe knee osteoarthritis (KOA) before and after a home exercise program (HEP) compared to healthy controls (CON). ** *p* < 0.01, *** *p* < 0.001.

RPTD of leg extensor muscles at 50% of MVC force increased (*p* < 0.01, CES 0.42) in the KOA group post-HEP compared to pre-HEP but was lower in both pre-HEP (*p* < 0.001) and post-HEP (*p* < 0.001) compared to the CON group (Figure 3).

### 3.3. Knee Range of Motion and Pain

Knee AROM flex of the involved limb increased (*p* < 0.01, CES = 0.48) post-HEP, yet was smaller both pre-HEP (*p* < 0.001) and post-HEP (*p* < 0.01) compared to the uninvolved limb and compared to the CON group’s dominant limb (*p* < 0.001) (Table 3). The KOA group demonstrated an extension deficit in the involved knee joint, yet no significant changes (*p* > 0.05, CES = 0.05) were observed post-HEP. The extension deficit of the KOA groups’ involved limb was higher (*p* < 0.01) compared to the uninvolved limb and compared to the CON group (*p* < 0.01) in both pre-HEP and post-HEP.

Self-reported knee pain (VAS) decreased post-HEP in the KOA group compared to pre-HEP, yet the difference was not statistically significant (*p* > 0.05, CES = 0.12) (Table 3).

### 3.4. Timed Up and Go Test

The TUG test result did not change significantly (*p* > 0.05, CES = 0.01) in the KOA group post-HEP (Table 3). The KOA group performed the test slower compared to the CON group pre-HEP (*p* < 0.05) but not post-HEP (*p* > 0.05).

### 3.5. Self-Reported Knee OA-Related Health Status

For better interpretation and comparison, the WOMAC score was converted and analyzed on a scale of 0–100, with higher scores reflecting better health status (Table 3). The KOA group’s WOMAC total index increased significantly (*p* < 0.05, CES = 0.52) post-HEP compared to pre-HEP. Pain reduced post-HEP, as indicated by a significantly higher WOMAC pain subscale score (*p* < 0.05, CES = 0.24). The clinical effect size indicated a clinically relevant increase in the function subscale (CES 0.39); however, this increase was not statistically significant (*p* > 0.05). The knee stiffness subscale remained unchanged (*p* > 0.05, CES = 0.04). All subscales and the WOMAC total index were smaller in the KOA group (*p* < 0.001) than in the CON group, both pre-HEP and post-HEP.

### 3.6. Correlations

Correlation coefficients between measured characteristics are shown in Table 4. Pre-HEP, RPTD (leg extensor muscle strength) was positively correlated (*p* < 0.05, r = 0.47) with KEM and negatively correlated (*p* < 0.01, r = −0.61) with step width. WOMAC subscales and total index were negatively correlated with VAS (*p* < 0.05, r = −0.51 − −0.69) and positively correlated with knee AROM flex (*p* < 0.05, r = 0.51–0.59) and RPTD (*p* < 0.05, r = 0.55–0.62). VAS and knee AROM flex were also negatively correlated (*p* < 0.01, r = −0.61) with each other and AROM flex negatively (*p* < 0.05, r = −0.57) with TUG.

Correlations between VAS and WOMAC (*p* < 0.01, r = −0.68–0.81) and between AROM flex and TUG (*p* < 0.01, r = −0.76) were maintained post-HEP. In addition, AROM flex correlated with spatiotemporal parameters of gait post-HEP, specifically, with step length (*p* < 0.01, r = 0.65), stride length (*p* < 0.001, r = 0.71), step width (*p* < 0.05, r = −0.52), and mean gait velocity (*p* < 0.05, r = 0.57).

## 4. Discussion

The results of the present study partially supported the first and second hypothesis/research question. The main results in the present study suggested that eight weeks of preoperative HEP in women with stages III–IV knee OA (by Kellgren and Lawrence) scheduled for TKA: (1) improves GRF and KEM during the loading response of gait, but not spatiotemporal characteristics; (2) increases leg extensor muscle strength and knee AROM; and (3) improves self-reported knee OA-related health status. Compared to the controls, the KOA group demonstrated similar results in KEM during the loading response post-HEP, but other measured characteristics were lower both pre-HEP and post-HEP. The third hypothesis was supported in the most part, as (1) gait characteristics were found to be associated with leg extensor muscle strength and knee AROM, but not pain, and (2) the self-reported knee OA-related health status measured with WOMAC was associated with leg extensor muscle strength, knee AROM, and pain. While previous research has focused primarily on patients with mild-to-moderate knee OA, this study adds value by providing insight into preoperative home-based rehabilitation in patients with severe knee OA who are undergoing TKA.

In this study, GRF of the involved limb improved 19.3% during the loading response of gait post-HEP, along with KEM increasing 37.3% and achieving similar results to the CON group post-HEP while being lower than the CON group pre-HEP. Fukaya et al. [19] have also found that early stance phase KEM is decreased in knee OA patients, especially in relation to the severity of symptoms, with more substantial pain leading to a smaller KEM. Based on the results of this study, an 8-week HEP can improve gait function in women with severe knee OA. It has been found that patients with knee OA have reduced KEM in the early stance phase, which tends to worsen as OA and its symptoms progress [20]. Therefore, although KEM did not improve significantly when comparing pre-HEP to post-HEP, it did improve, as indicated by the KOA group reaching the level of the CON group. This suggests enhanced gait efficiency and movement control in relation to gravity during the stance phase of gait [67]. Also, GRF in the KOA group showed less asymmetry between limbs after the HEP, indicating a more symmetrical gait. This improvement towards symmetry is noteworthy, as vertical GRF contributes to KEM alterations during gait [68]. Therefore, improvement of GRF could have been one of the reasons for the improvement in KEM to the CON group level. However, other gait-related characteristics, like the TUG test and gait spatiotemporal parameters, did not change post-HEP and remained inferior to the CON group. Previous studies have also found that knee OA patients have slower walking speed, especially in the later stages of the disease, yet exercise therapy has been found to be effective in improving walking speed in knee OA patients [20,41,69]. As patients with stages III–IV knee OA have been living with the diagnosis for an average of more than 13 years before receiving TKA and have moderate-to-severe structural changes hindering improvement in function [70,71], it can be assumed that an eight-week HEP is not long enough to have effects on such aspects of gait.

There could also be other associated factors besides GRF. Previous studies have found associations between knee moment and knee pain, stating that knee OA patients have reduced knee moments in an attempt to reduce pain during gait, and more pain leads to a smaller KEM [19,55]. In this study, no associations were observed between knee pain (VAS) and gait characteristics, although VAS decreased 13.2%, and the WOMAC pain score improved in the KOA group post-HEP, suggesting that perceived pain could have potentially influenced the KEM and GRF.

The present study also found a clinically and statistically relevant 40% increase in the leg extensor muscle RPTD in the KOA group post-HEP, which was positively associated with KEM and another possible reason for its improvement to the CON group level. The knee extensors are the primary muscles activated during the loading response of gait [21,72], and the present study showed that the leg extensor muscles RPTD and KEM were positively related pre-HEP. This association indicates that lower muscle strength contributed to a lower KEM pre-HEP, which refers to greater muscle strength promoting higher KEM and more weight loaded on the involved limb during the loading response post-HEP. As a result, the gait is more stable and efficient, as also confirmed by the increased GRF and reduced GRF asymmetry between limbs in the KOA group post-HEP.

Li et al. [17] reported gait analysis to be significantly associated with the WOMAC score. The present study found no association between gait characteristics and the self-reported knee OA-related health status. Instead, improved WOMAC was associated with increased RPTD. As RPDT improved post-HEP, the WOMAC pain and function subscales and total index also showed statistically and clinically relevant improvements (20%, 17%, and 18%, respectively). Therefore, it can be assumed that improved leg extensor muscle strength subsequently influenced KOA groups’ improved self-reported knee OA-related health status post-HEP. Thus, it supports the idea that exercise interventions in this patient population should include resistance training and targeting leg extensor muscle strength. O’Reilly et al. [70] noted similar improvements in the WOMAC pain and function subscales (22.5% and 17.4%, respectively) after a HEP, attributing these benefits to muscle strength gains improving the self-reported knee OA-related health status. Similarly, Kim et al. [73] identified knee extensors as a significant factor influencing the WOMAC score.

In the present study, another factor influencing the self-reported knee OA-related health status was VAS-measured knee pain, indicating that pain should also be one of the focuses in preoperative HEPs, as a lower pain level post-HEP was also a promoter to the improved WOMAC scores post-HEP. This finding corresponds with a study by Aytekin et al. [50], who state that about 20% of their patients in the prehabilitation group postponed TKA due to decreased pain. In this study, improved self-rated knee OA-related health status and increased muscle strength and knee function may have been reasons for postponed TKA surgery in five women (27.8%) in the KOA group. Post-HEP knee function improved as the knee AROM flex increased in the KOA group. Although the KOA group had lower knee AROM flex than the CON group, their AROM flex increased by 9.7% post-HEP. In turn, the improvement of knee AROM flex could have also contributed to improved WOMAC scores, as these characteristics were found to be associated.

Knee AROM can also affect gait characteristics [74]. Although the current study noted associations between knee AROM and gait characteristics, the spatiotemporal characteristics remained unchanged post-HEP, despite increased knee AROM flex. This might have occurred due to the length of the intervention period and the stage of the knee OA, as complications and symptoms of end-stage knee OA are more profound [20]. Research with longer preoperative HEP is needed to see its potential effect on spatiotemporal characteristics in patients with severe knee OA.

This study has limitations, as due to the small sample size, the results may not be fully generalizable to the broader knee OA or healthy population; however, they provide valuable insights into observed trends, highlighting the potential benefits of the intervention and warranting larger follow-up studies. Another limitation was that only women were included; therefore, applying this knowledge to the general age-matched population is problematic. Also, the KOA group participants had a significantly higher body mass and BMI than the CON group. However, knee OA is associated with higher body mass in women, likely connected to lower physical activity. Thus, OA patients tend to be less physically active due to their symptoms [75,76]. In the HEP, standardized resistance bands were used; however, the applied force was not objectively measured. Also, the postoperative effects of the HEP were not assessed in the present article, which would have allowed for an assessment of whether the improvements achieved before surgery translated into subsequent benefits. Lastly, body composition was not assessed.

However, findings regarding the effect of a preoperative HEP on gait characteristics and the self-reported knee OA-related health status in women with severe knee OA scheduled for TKA may assist orthopedic surgeons, physical therapists, and other healthcare professionals in planning preoperative rehabilitation and choosing treatment options. Considering the need for prevention and treatment in patients with OA, especially with the prevalence of knee OA and joint replacement surgeries rising, more research on preoperative home exercise programs is needed to analyze their effect on different aspects of prehabilitation, especially in patients with severe knee OA.

## 5. Conclusions

An 8-week preoperative HEP focusing on resistance, balance, coordination, and stretching exercises was beneficial for women with severe knee OA scheduled for arthroplasty for improving gait function (GRF and KEM) during the loading response of gait, increasing leg extensor strength and knee function, and consequently improving patients’ self-rated knee OA-related health status. Gait and the self-reported knee OA-related health status were mainly associated with leg extensor muscle strength, knee AROM, and pain. Consideration should be given to focusing on these aspects in preoperative rehabilitation and home interventions in patients with severe knee OA. Future studies should examine body composition characteristics and their relationship with functional outcomes to clarify further the reasons for the improvement in function and knee OA-related health status.

## Figures and Tables

**Table 1:** Data of participants.

Parameters	KOA		CON
	Pre-HEP	Post-HEP	
N	18	18	10
Age (years)	61.8 ± 1.6	62.0 ± 1.6	62.1 ± 1.8
Weight (kg)	88.8 ± 3.8 **	88.5 ± 3.7 **	70.6 ± 4.1
Height (m)	161.8 ± 1.2	161.8 ± 1.2	161.1 ± 1.8
BMI (kg/m^2^)	33.9 ± 1.4 **	33.9 ± 1.4 **	27.2 ± 1.3

Data are means ± SE; BMI—body mass index; CON—control group; HEP—home exercise program; KOA—knee osteoarthritis group; N—number of participants; ** *p* < 0.01 compared to controls.

**Table 2:** Spatiotemporal characteristics of gait in knee OA patients before and after a home exercise program, and in healthy controls.

Characteristic	KOA		CON
	Pre-HEP	Post-HEP	
Stance time (%)	58.99 ± 0.55 *	58.95 ± 0.43 *	57.33 ± 0.36
Cadence (steps/min)	107.36 ± 2.01 ***	111.41 ± 2.62 **	124.41 ± 2.86
Mean velocity (m/s)	1.12 ± 0.04 ***	1.12 ± 0.06 ***	1.46 ± 0.04
Stride length (m)	1.22 ± 0.03 ***	1.23 ± 0.04 **	1.41 ± 0.03
Step length (m)	0.62 ± 0.02 ***	0.61 ± 0.02 ***	0.72 ± 0.02
Step width (m)	0.08 ± 0.01	0.10 ± 0.01	0.10 ± 0.01

Data are mean ± SE; CON—control group, HEP—home exercise program, KOA—knee osteoarthritis group; * *p* < 0.05, ** *p* < 0.01, *** *p* < 0.001 compared to controls.

**Table 3:** Functional performance and self-reported knee OA-related health status in women with severe knee osteoarthritis before and after a home exercise program, and in healthy controls.

Characteristic		KOA		CON
		Pre-HEP	Post-HEP	
WOMAC	Pain	11.33 ± 1.18 ^###^	13.56 ± 0.97 **^,###^	19.50 ± 0.22
	CI 95%	10.79–11.88	13.11–14.00	19.36–19.64
	Stiffness	4.17 ± 0.55 ^###^	4.44 ± 0.55 ^###^	8.00 ± 0.00
	CI 95%	3.91–4.42	4.19–4.70	8.00–8.00
	Function	37.83 ± 3.71 ^###^	44.33 ± 3.07 ^###^	67.40 ± 0.34
	CI 95%	36.12–39.55	42.91–45.75	98.51–99.19
	total index	55.55 ± 5.28 ^###^	65.74 ± 4.66 *^,###^	98.85 ± 0.55
	CI 95%	53.11–57.99	63.59–67.89	98.51–99.19
VAS (points)		5.14 ± 0.42 ^###^	4.47 ± 0.45 ^###^	0.2 ± 0.15
	CI 95%	4.95–5.34	4.26–4.67	0.11–0.29
AROM flex (◦)	involved	90.9 ± 4.6 ^###,&&&^	99.8 ± 3.8 **^,###,&&^	122.8 ± 2.0
	uninvolved	108.9 ± 2.3	113.8 ± 1.9	N/A
AROM ext (◦)	involved	−7.5 ± 2.0 ^##,&&^	−6.9 ± 1.7 ^##,&&^	−0.1 ± 0.1
	uninvolved	−0.5 ± 0.4	0.0 ± 0.0	N/A
TUG (s)		9.05 ± 0.99 ^#^	9.00 ± 1.20	5.94 ± 0.27

Data are means ± SE; AROM flex—active knee flexion range of motion; AROM ext—active knee extension range of motion; CI 95%—confidence intervals at 95%; CON—control group; HEP—home exercise program; KOA—knee osteoarthritis group; TUG—Timed Up and Go test; VAS—visual analogue scale (0–10 points); WOMAC—Western Ontario and McMaster Universities Osteoarthritis Index; * *p* < 0.05 and ** *p* < 0.01 pre-HEP compared to post-HEP; ^&&^ *p* < 0.01; ^&&&^ *p* < 0.001 involved limb compared to uninvolved limb; ^#^ *p* < 0.05, ^##^ *p* < 0.01; ^###^ *p* < 0.001 compared to the controls.

**Table 4:** Pre-HEP and post-HEP correlation coefficients between functional, gait, and self-reported characteristics in women with severe knee osteoarthritis.

	Pre-HEP			Post-HEP	
	RPTD	AROM Flex	VAS	AROM	VAS
KEM	0.47 *	−0.27	0.15	−0.15	0.26
Step length	0.21	0.33	−0.46	0.65 **	−0.13
Stride length	0.20	0.15	−0.34	0.71 ***	−0.22
Step width	−0.61 **	−0.14	0.36	−0.52 *	0.30
Mean gait velocity	0.15	0.07	−0.40	0.57 *	−0.19
WOMAC pain	0.55 *	0.39	−0.51 *	0.26	−0.81 ***
WOMAC stiffness	0.44	0.51 *	−0.56 *	0.63 **	−0.68 **
WOMAC function	0.61 **	0.59 **	−0.69 ***	0.27	−0.81 ***
WOMAC total index	0.62 **	0.58 **	−0.68 **	0.31	−0.79 ***
AROM flex	0.24	N/A	−0.61 **	N/A	−0.41
TUG	−0.13	−0.57 *	0.07	−0.76 ***	0.24

AROM flex—active knee flexion range of motion, pre-HEP—before the home exercise program; post-HEP—after the home exercise program, KEM—knee extension moment, RPTD—relative peak torque development, TUG—Timed Up and Go test, VAS—visual analogue scale, WOMAC—Western Ontario and McMaster Universities Osteoarthritis Index. * *p* < 0.05, ** *p* < 0.01, *** *p* < 0.001.

## Data Availability

The data supporting the results of this study are available from the corresponding author upon reasonable request.

## Appendix A

**Table A1:** Preoperative 8-Week Home Exercise Program for Patients with Severe Knee Joint Osteoarthritis Scheduled for Total Knee Arthroplasty.

Exercises	Progression	Description
Ex. 1		Walk at a medium pace, hands move along the sides.
Stationary walking	1–8 weeks	1 repetition of 3 min.
Ex. 2		While walking, do knee lifts as high as possible. Walk at a medium pace, hands move along the sides.
High knee walking	1–8 weeks	1 repetition of 1 min.
Ex. 3		While sitting, place a ball between your knees and press the ball with the knees for 2–3 s and then relax.
Hip adductors strength	1–2 weeks	1 set of 16 repetitions at low resistance.
	3–4 weeks	2 sets of 16 repetitions at low resistance.
	5–6 weeks	1 set of 16 repetitions at moderate resistance.
	7–8 weeks	2 sets of 16 repetitions at moderate resistance.
Ex. 4		While sitting, tie a rubber band around your knees and push your knees outward against the rubber band. Exertion held for 2–3 s and then relaxed.
Hip abductors strength	1–2 weeks	1 set of 10–16 repetitions at low resistance.
	3–4 weeks	2 sets of 10–16 repetitions at low resistance.
	5–6 weeks	1 set of 10–16 repetitions at moderate resistance.
	7–8 weeks	2 sets of 10–16 repetitions at moderate resistance.
Ex. 5		While sitting, straighten the lower limb against the rubber band placed under the foot. Hold for 2–3 s, relax. Do the exercise alternately.
Knee extensor strength	1–2 weeks	1 set of 16 repetitions at low resistance.
	3–4 weeks	2 sets of 16 repetitions at low resistance.
	5–6 weeks	1 set of 16 repetitions at moderate resistance.
	7–8 weeks	2 sets of 16 repetitions at moderate resistance.
Ex. 6		Sit on the chair. Make 3 “attempts” to get up from the chair (without actually getting up). Get up on the 4th time.
Leg extensor strength, balance	1–2 weeks	1 set of 12 repetitions at low resistance.
	3–4 weeks	2 sets of 12 repetitions at low resistance.
	5–6 weeks	1 set of 12 repetitions at moderate resistance.
	7–8 weeks	2 sets of 12 repetitions at moderate resistance.
Ex. 7		Stand behind the chair, hold the backrest. Rise on your toes and hold the position for 2–3 s.
Dorsiflexor strength, balance	1–2 weeks	1 set of 20 repetitions at low resistance.
	3–4 weeks	2 sets of 20 repetitions at low resistance.
	5–6 weeks	1 set of 20 repetitions at moderate resistance.
	7–8 weeks	2 sets of 20 repetitions at moderate resistance.
Ex. 8		Stand behind the chair, hold the backrest. Stand on your heels and hold the position for 2–3 s.
Plantarflexor strength, balance	1–2 weeks	1 set of 20 repetitions at low resistance.
	3–4 weeks	2 sets of 20 repetitions at low resistance.
	5–6 weeks	1 set of 20 repetitions at moderate resistance.
	7–8 weeks	2 sets of 20 repetitions at moderate resistance.
Ex. 9		Stand behind the chair, hold the backrest. Move your straight leg to the side. Toes pointed forward. Do the exercise alternately.
Gluteus maximus strength, balance	1–2 weeks	1 set of 20 repetitions at low resistance.
	3–4 weeks	2 sets of 20 repetitions at low resistance.
	5–6 weeks	1 set of 20 repetitions at moderate resistance.
	7–8 weeks	2 sets of 20 repetitions at moderate resistance.
Ex. 10		Stand behind the chair, hold the backrest. Start squatting until you reach a semi-squat. Make sure your knees do not go over the line of your toes.
Knee flexors and extensors strength	1–2 weeks	1–2 sets of 16 repetitions at low resistance.
	3–4 weeks	1–2 sets of 16 repetitions at low resistance.
	5–6 weeks	2–3 sets of 16 repetitions at moderate resistance.
	7–8 weeks	2–3 sets of 16 repetitions at moderate resistance.
Ex. 11		Stand on one foot as long as possible. Repeat with other side.
Balance	1–2 weeks	1 set of 8 repetitions at low resistance.
	3–4 weeks	2 sets of 8 repetitions at low resistance.
	5–6 weeks	1 set of 8 repetitions at moderate resistance.
	7–8 weeks	2 sets of 8 repetitions at moderate resistance.
Ex. 12		While sitting, extend one leg forward and place it on the heel. Bend forward to feel the stretch in the back of your thigh. Perform the exercise alternately and with maximally tolerable knee extension. Hold for 6–8 s.
Hamstring stretch	1–2 weeks	1 set of 16 repetitions at low resistance.
	3–4 weeks	2 sets of 16 repetitions at low resistance.
	5–6 weeks	1 set of 16 repetitions at moderate resistance.
	7–8 weeks	2 sets of 16 repetitions at moderate resistance.
Ex. 13		While sitting, extend one leg forward and place it on the heel. Bend forward to feel the stretch in the back of your thigh. Perform the exercise alternately and with maximally tolerable knee extension and ankle dorsiflexion. Hold for 6–8 s.
Hamstring stretch	1–8 weeks	1 set of 6 repetitions.
Ex. 14		While sitting, extend one leg forward and place it on the heel. Pull your toes towards you as much as possible. Perform the exercise alternately and with maximally tolerable ankle dorsiflexion. Hold for 6–8 s.
Gastrocnemius stretch	1–8 weeks	1 set with 6 repetitions.
Ex. 15		Rest lying down.
Relaxation	1–8 weeks	Breathe deeply and relax.

Low resistance—using red elastic band, moderate resistance—using green and then blue elastic band (Theraband, Akron, OH, USA).
